# Overexpression of the Jojoba Aquaporin Gene, *ScPIP1*, Enhances Drought and Salt Tolerance in Transgenic *Arabidopsis*

**DOI:** 10.3390/ijms20010153

**Published:** 2019-01-03

**Authors:** Xing Wang, Fei Gao, Jie Bing, Weimin Sun, Xiuxiu Feng, Xiaofeng Ma, Yijun Zhou, Genfa Zhang

**Affiliations:** 1 Beijing Key Laboratory of Gene Resource and Molecular Development, College of Life Sciences, Beijing Normal University, Beijing 100875, China; xingwang163@163.com (X.W.); bingjie@bnu.edu.cn (J.B.); 201021200018@bnu.edu.cn (W.S.); 15249220644@163.com (X.F.); mxf510332934@163.com (X.M.); 2 College of Life and Environmental Sciences, Minzu University of China, Beijing 100081, China; gaofei@muc.edu.cn

**Keywords:** *Simmondsia chinensis*, aquaporin, PIP1, drought stress, salt stress

## Abstract

Plasma membrane intrinsic proteins (PIPs) are a subfamily of aquaporin proteins located on plasma membranes where they facilitate the transport of water and small uncharged solutes. PIPs play an important role throughout plant development, and in response to abiotic stresses. Jojoba (*Simmondsia chinensis* (Link) Schneider), as a typical desert plant, tolerates drought, salinity and nutrient-poor soils. In this study, a *PIP1* gene (*ScPIP1*) was cloned from jojoba and overexpressed in *Arabidopsis thaliana*. The expression of *ScPIP1* at the transcriptional level was induced by polyethylene glycol (PEG) treatment. *ScPIP1* overexpressed *Arabidopsis* plants exhibited higher germination rates, longer roots and higher survival rates compared to the wild-type plants under drought and salt stresses. The results of malonaldehyde (MDA), ion leakage (IL) and proline content measurements indicated that the improved drought and salt tolerance conferred by *ScPIP1* was correlated with decreased membrane damage and improved osmotic adjustment. We assume that *ScPIP1* may be applied to genetic engineering to improve plant tolerance based on the resistance effect in transgenic *Arabidopsis* overexpressing *ScPIP1*.

## 1. Introduction

Aquaporins transport water, CO_2_ and small neutral solutes through the plasma and intracellular membranes of cells [1,2,3]. They play a central role in regulating water transport in many physiological and developmental processes, including cell elongation, stomatal regulation, seed germination, reproductive growth and stress responses in plants [4,5]. Based on conserved amino acid sequences and intron positions, AQPs are divided into five groups: plasma membrane intrinsic proteins (PIPs), tonoplast intrinsic proteins (TIPs), nodulin-like plasma membrane intrinsic proteins (NIPs), small intrinsic proteins (SIPs) and X intrinsic proteins (XIPs) [6]. The plasma membrane intrinsic proteins present in the plasma membrane regulate water movement between cells and coordinate with TIPs to maintain cell water balance.

Drought, high salinity, low temperature and other abiotic stresses could result in water loss in plants, which seriously limits plant growth, development and productivity worldwide. Water movement is a key physiological process of plants and must be tightly regulated under drought and salt stress conditions [7,8,9]. The transport of water is controlled by both symplastic and apoplastic pathways [10]. The symplastic pathway in which aquaporins (AQPs) play a central role is efficient for transporting water across membranes [11,12,13,14].

The expression regulation, biological activity and localization of *PIPs* are regulated by abiotic stresses, plant hormones and light [15,16,17,18,19,20]. *Arabidopsis* PIP transcripts are generally downregulated upon gradual drought stress in leaves, with the exception of *AtPIP1;4* and *AtPIP2;5*, which are upregulated [21]. *AtPIP2;6* and constitutively expressed and not significantly affected by the drought stress [21]. Under drought stress, the accumulation of *NtPIP1;1* and *NtPIP2;1* transcripts was significantly decreased, but only that of the *NtAQP1* transcript was increased [22]. The level of *VfPIP1* mRNA had obviously increased from 0.5 to 2.0 h after 20% PEG6000PEG treatment [23]. During 100 mmol/L NaCl treatment, *GmPIP1;6* reduced expression initially, then the expression was increased in root and leaves after 3 days [24]. *OsPIP1;3* was induced by 2.5-fold at 2 h of 250 mmol/L NaCl treatment and then gradually declined; whereas the expression of *OsPIP1;1* increased by about two-fold constantly [25].

By means of transgenic approaches, some studies have demonstrated that overexpressing a *PIP1* gene in plants confers tolerance to abiotic stresses based on the roles of PIP1s in response to the adverse environment [23,24,25,26,27,28]. The overexpression of *TaAQP7* in tobacco enhanced drought tolerance [27], and the overexpression of *TaAQP8*, another wheat *PIP* gene, induces salt stress tolerance in tobacco when compared with wild-type plants [28]. However, not all *PIPs* confer tolerance to abiotic stresses in transgenic plants. The overexpression of *HvPIP2;1* resulted in the transgenic rice being more sensitive to drought and salt stresses because of an increased transpiration rate and decreased intrinsic water-use efficiency [29]. Previous studies have demonstrated that the different effects of overexpressing *PIPs* depended on the sources and isoforms of these genes. Therefore, specific *PIPs*, which confer plants better growth and development status under abiotic stresses, are potential genetic resources in agronomy and crop science.

However, there is no research on the overexpression of the *PIP1* gene from desert plants, which have many precious agronomic traits. Jojoba, as a typical desert plant, has very rare agronomic traits, such as tolerance to high-temperature, drought, salt and poor soil environments. Exploring germplasm resources, cloning stress response genes and researching gene functions will have important value. *ScPIP1* is a drought-induced gene, suggesting its role in response to water deficit, and there are no reports on this gene so far. In this study, we first cloned the full-length *ScPIP1* gene encoding a plasma membrane intrinsic protein in Jojoba. Based on a transgenic approach, *ScPIP1* overexpressing *Arabidopsis* plants were generated, and their resistance was evaluated at physiological and biochemical levels. Additionally, the cause of improved tolerance was reported in our study.

## 2. Results

### 2.1. ScPIP1 Gene Encodes a PIP1 Subgroup of AQPs in Jojoba

**Figure 1:**
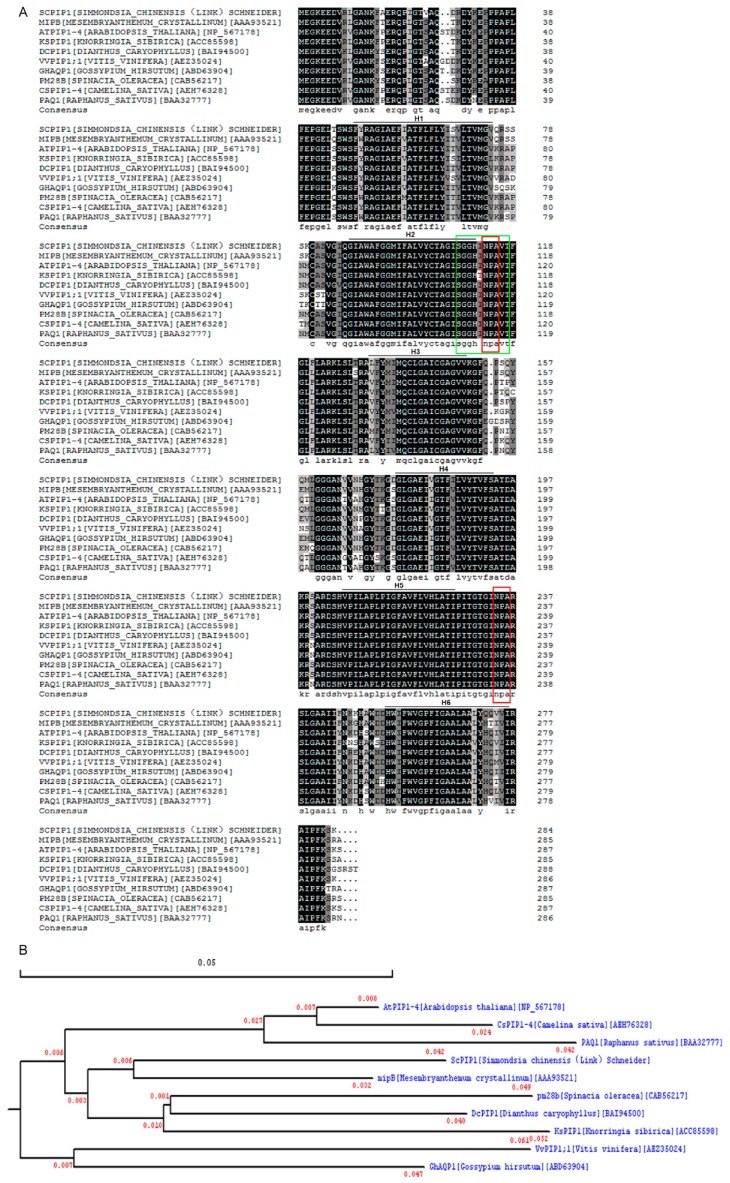
Alignment and phylogenetic analysis of ScPIP1 with other known PIP proteins. Amino acid sequences were aligned by DNAMAN software (**A**). The conserved amino acid residues in all proteins are highlighted in grey. Two conserved NPA motifs (red frame) and the MIP signal sequence (green frame) are boxed. The six putative transmembrane α-helices (H1–H6) are shown by lines. Phylogenetic analysis of ScPIP1 was conducted by the maximum likelihood method (**B**). Values at nodes in the phylogenetic tree in Figure 1B represent bootstrap values indicating branching probability per 1000 replicates.

Our laboratory has screened 385 unique ESTs based on a water stress induced suppression subtractive hybridization (SSH) cDNA library of jojoba. We found that the EST sequence (Genbank accession number: DV752738) of aquaporin was highly repeated, indicating that this gene was regulated by water deficit [30]. By using the rapid amplification of cDNA ends (RACE) method, a full-length cDNA of the aquaporin-like gene was isolated from cDNA prepared from jojoba leaves and designated as *ScPIP1*. Analysis of the sequence showed that *ScPIP1* cDNA is 1244 bp in length and contains a 5’-noncoding region (74 bp) and an open reading frame (855 bp) that encodes 284 amino acids and a 3’-noncoding region (315 bp). The analysis of the amino acid sequence revealed that *ScPIP1* had 93% sequence identity with mipB of *Mesembryanthemum crystallinum*. ScPIP1 contains six putative transmembrane α-helices, two ‘NPA’ motifs and an MIP signal sequence (SGxHxNPAVT) in loop B (Figure 1). Phylogenetic analysis of ScPIP1 with mipB [*M. crystallinum*] (AAA93521), AtPIP1;4 [*A. thaliana*] (NP_567178), KsPIP1 [*Knorringia sibirica*] (ACC85598), DcPIP1 [*Dianthus caryophyllus*] (BAI94500), VvPIP1;1 [*Vitis vinifera*] (AEZ35024), GhAQP1 [*Gossypium hirsutum*] (ABD63904), pm28b [*Spinacia oleracea*] (CAB56217), CsPIP1-4 [*Camelina sativa*] (AEH76328) and PAQ1 [*Raphanus sativus*] (BAA32777) showed that ScPIP1 and mipB belong to the same class. These results suggest that the *ScPIP1* gene isolated in this study is a member of the *PIP1* subfamily in Jojoba.

### 2.2. ScPIP1 Is Upregulated in Jojoba Leaves after PEG Treatment

**Figure 2:**
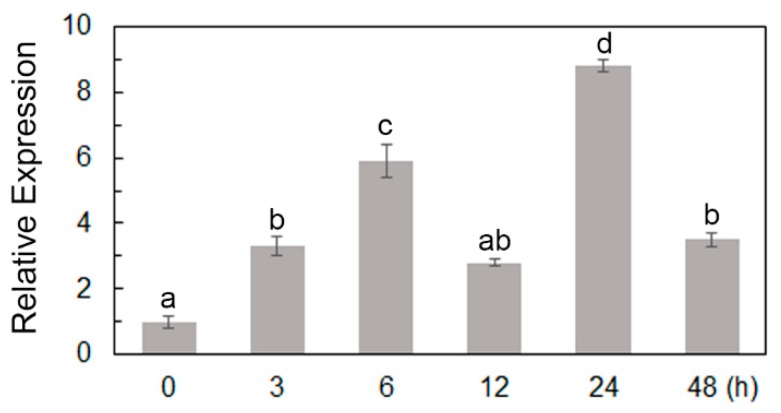
Regulation of *ScPIP1* expression under PEG stress. Characterization of the qRT-PCR analysis of *ScPIP1* from jojoba plants treated with 30% PEG6000 for 0, 3, 6, 12, 24 or 48 h. The data are shown as the means ± standard deviation (SD) of 3 biological replicates. Significant differences were determined by one-way ANOVA followed by Duncan’s multiple range test at *p* < 0.05.

To investigate the expression mode of *ScPIP1* in jojoba leaves under PEG stress, six-week old jojoba seedlings were treated with 30% PEG6000 for 0, 3, 6, 12, 24 or 48 h and total RNA was extracted from jojoba leaves. Moreover, mRNA was converted to cDNA before being subjected to real-time quantitative polymerase chain reaction (qRT-PCR) analysis. *ScPIP1* transcripts were steadily upregulated within 6 h and downregulated at 12 h. The highest expression levels appeared at 24 h, however, the expression of *ScPIP1* transcripts reduced at 48 h (Figure 2).

### 2.3. Phenotypic Analysis of Transgenic Arabidopsis Lines Overexpressing ScPIP1

**Figure 3:**
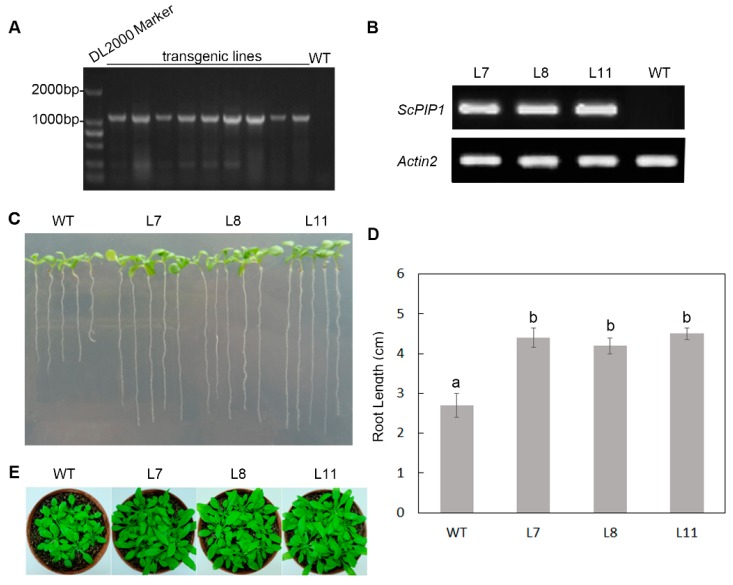
Molecular and phenotype analysis of *ScPIP1* overexpressing Arabidopsis lines. Genomic DNA PCR analysis (**A**) of WT and transgenic lines. Semiquantitative RT-PCR characterization (**B**) of *ScPIP1* and *AtActin2* in WT and transgenic lines. Images (**C**) and the results of statistical analyses (**D**) of the root lengths of nineteen-day-old WT and transgenic lines. (**E**) Images of four-week-old transgenic lines and WT under optimum growth conditions. The data are presented as the means ± SD of 3 biological replicates. Significant differences were determined by one-way ANOVA followed by Duncan’s multiple range test at *p* < 0.05.

To determine the role of *ScPIP1* in plants, *ScPIP1* was transformed into the pCAMBIA1302 vector under the control of the 35S promoter. Through the floral-dip transformation of *Arabidopsis*, three homozygous T_3_ transgenic lines (L7, L8 and L11) were selected on MS medium containing 50 mg/L hygromycin. *ScPIP1* overexpressing transgenic lines L7, L8 and L11 exhibited longer root lengths and better growth status than the wild type (WT) (Figure 3A–E). These results suggest that the overexpression of *ScPIP1* influences root elongation and leaf development under optimum growth conditions.

### 2.4. Overexpression of ScPIP1 in Arabidopsis Enhances Tolerance to Drought Stress

**Figure 4:**
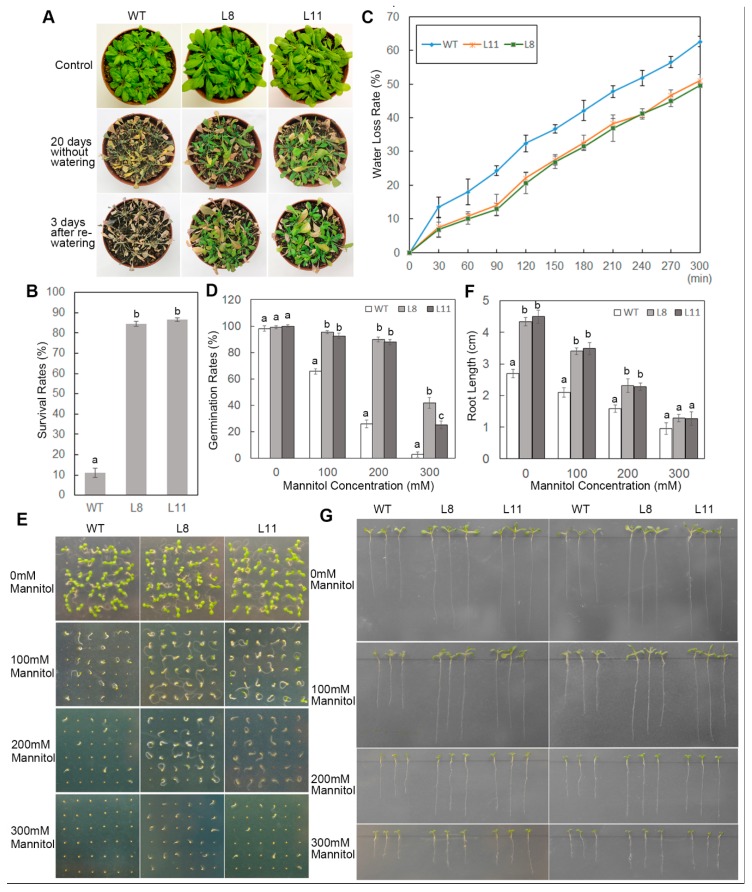
Phenotypes of *ScPIP1* overexpressing transgenic lines under drought. Images (**A**) and survival rates (**B**) of the four-week-old WT and transgenic lines under drought stress for 20 days. (**C**) Water loss rates of the leaves of WT and transgenic lines in vitro. Results of the statistical analyses (**D**) and images (**E**) of the germination test of the WT and transgenic lines under mannitol treatments for 5 days. Results of the statistical analyses (**F**) and images (**G**) of the root lengths of the WT and transgenic lines under mannitol treatments for 15 days. The data are presented as the means ± SD of 3 biological replicates. Significant differences were determined by one-way ANOVA followed by Duncan’s multiple range test at *p* < 0.05.

To investigate the drought tolerance of transgenic *Arabidopsis* overexpressing *ScPIP1*, four-week-old WT and transgenic *Arabidopsis* were treated without watering for 20 days and then rewatered. The transgenic lines exhibited higher survival rates than the WT (Figure 4A,C). The transgenic *Arabidopsis* had a lower water loss rate (Figure 4B). To determine the role of *ScPIP1* in transgenic *Arabidopsis* under drought stress, WT and transgenic seeds and seedlings were treated with mannitol. The transgenic lines showed higher germination rates and longer roots than the WT under mannitol treatment (Figure 4D–G). These results revealed that the transgenic lines exhibited better growth, higher germination rates, longer roots, higher survival rates and lower water loss rates compared to the WT under drought and osmotic stresses, indicating that the overexpression of *ScPIP1* improves the tolerance of *Arabidopsis* to drought and osmotic stresses.

### 2.5. Overexpression of ScPIP1 in Arabidopsis Decreases MDA Content and IL, and Increases Proline Accumulation under Drought Stress

**Figure 5:**
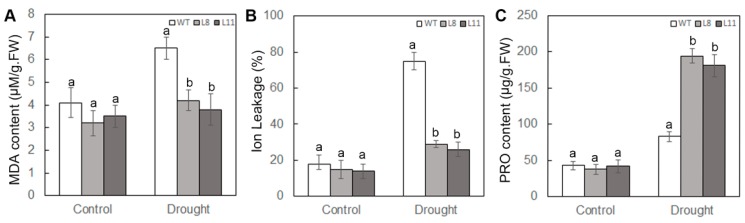
Analysis of the MDA, IL and proline contents of the WT and *ScPIP1* overexpressing transgenic lines under drought stress. The malonaldehyde content (**A**), ion leakage (**B**) and proline content (**C**) measured in the leaves of WT and transgenic lines that were well watered or without watered for 20 days. The data are presented as the means ± SD of 3 biological replicates. Significant differences were determined by one-way ANOVA followed by Duncan’s multiple range test at *p* < 0.05.

Increased drought and osmotic tolerance in the transgenic *Arabidopsis* compared to the WT allowed us to investigate the physiological and biochemical differences between WT and *ScPIP1* overexpressing transgenic lines. Malonaldehyde (MDA), ion leakage (IL) and proline content were quantified in the WT and *ScPIP1* overexpressing transgenic lines under optimum and drought conditions. The results revealed that there was no significant difference in MDA, IL and proline content between the WT and transgenic lines under optimum growth condition. However, compared to the WT, reduced MDA and IL, and higher proline content were observed in the transgenic lines under drought treatment (Figure 5A–C). These results indicate that the overexpression of *ScPIP1* reduces lipid peroxidation and maintains the accumulation of osmotic substances under drought stress.

### 2.6. Overexpression of ScPIP1 Enhances Tolerance to Salt Stress

**Figure 6:**
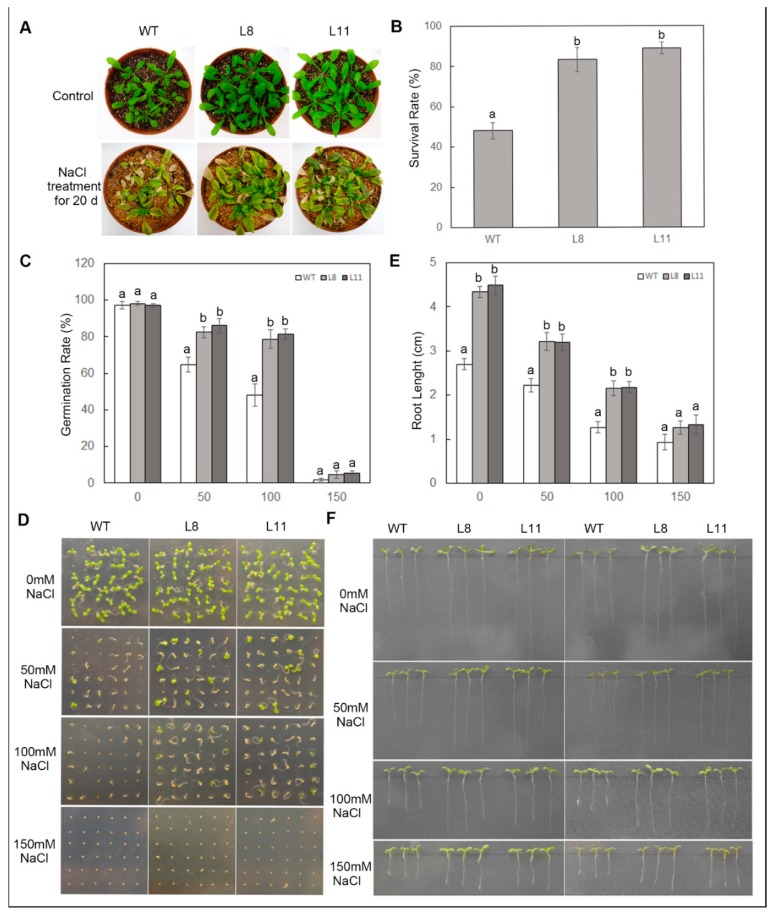
Phenotypes of *ScPIP1* overexpressing transgenic lines under salt stress. Images (**A**) and survival rates (**B**) of the four-week-old WT and transgenic lines under salt stress for 20 days. Results of the statistical analyses (**C**) and images (**D**) of the germination test of the WT and transgenic lines under NaCl treatments for 5 days. Results of the statistical analyses (**E**) and images (**F**) of the root lengths of the WT and transgenic lines under NaCl treatment for 15 days. The data are presented as the means ± SD of 3 biological replicates. Significant differences were determined by one-way ANOVA followed by Duncan’s multiple range test at *p* < 0.05.

To examine the role of *ScPIP1* during salt stress, WT and *ScPIP1* overexpressing transgenic lines were treated with NaCl. When four-week-old WT and *ScPIP1* overexpressing transgenic lines were treated with 300 mM NaCl for 20 d, *ScPIP1* overexpressing transgenic lines showed a higher survival rate and more green leaves compared to the WT (Figure 6A,B). To understand the role of *ScPIP1* in seed germination and root development, the WT and *ScPIP1* overexpressing transgenic lines were subjected to 50 mM to 150 mM NaCl treatment. The transgenic seeds exhibited higher germination rates and the transgenic seedlings showed longer root lengths than those of WT (Figure 6C–F). These results indicate that *ScPIP1* overexpressing transgenic lines showed better growth, higher survival rates, higher germination rates and longer roots than those of the WT plants. Therefore, *ScPIP1* overexpressing transgenic lines were more tolerant to salt stress than the WT.

### 2.7. Overexpression of ScPIP1 in Arabidopsis Decreases MDA Content and IL, and Increases Proline Accumulation under Salt Stress

**Figure 7:**
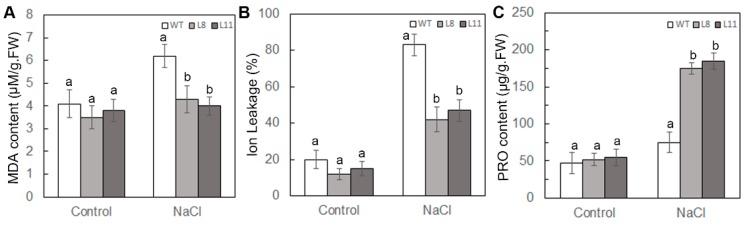
Analysis of the MDA, IL and proline contents of the WT and *ScPIP1* overexpressing transgenic lines under salt stress. Malonaldehyde content (**A**), ion leakage (**B**) and proline content (**C**) measured in the leaves of WT and transgenic lines under optimum and salt conditions. The data are presented as the means ± SD of 3 biological replicates. Significant differences were determined by one-way ANOVA followed by Duncan’s multiple range test at *p* < 0.05.

To study the role of *ScPIP1* in improving the salt tolerance of transgenic *Arabidopsis* compared to that in WT plants, the MDA, IL and proline contents were quantified in the WT and *ScPIP1* overexpressing transgenic lines under optimum and salt conditions. Similar to the drought stress experiments, there was no significant difference in the MDA, IL and proline contents between the WT and the transgenic lines under optimum growth conditions. However, under NaCl treatment, the transgenic lines exhibited reduced MDA and IL, and higher proline content compared to the WT (Figure 7A–C), suggesting less membrane damage caused by lipid peroxidation and more accumulation of osmotic substances in the transgenic lines.

## 3. Discussion

### 3.1. Overexpression of ScPIP1 Enhances Plant Resistance to Drought and Salt Stresses

It has been reported that PIPs are involved in abiotic stress resistance [3,5,25,26,27,28,29,30,31]. In the present study, we found that the expression level of *ScPIP1* in jojoba leaves was upregulated under PEG treatment, indicating that *ScPIP1* plays a positive role in response to osmotic and drought stresses. To further examine the function of *ScPIP1* under drought and salt stress, we generated three homozygous *ScPIP1* overexpressing transgenic *Arabidopsis* lines. The transgenic seeds, seedlings and adult plants were more tolerant to drought and salt stresses compared to the WT plants. Consistent with previous studies [15,16,21,22,23,24,25], these results demonstrated that overexpression of some specific *PIP* genes enhances abiotic stress resistance.

### 3.2. Response of ScPIP1 to Abiotic Stress Involved in Reducing Membrane Damage

Drought and salt stresses cause the rapid accumulation of reactive oxygen species (ROS), resulting in membrane damage and oxidation [32,33]. MDA, a product of lipid peroxidation caused by ROS, can be used to evaluate ROS induced damage in plants [34]. Ion leakage is also a significant indicator of membrane damage. Therefore, MDA content and IL were measured to examine the role of *ScPIP1* in decreasing the membrane damage caused by lipid peroxidation under optimum, drought or salt conditions. The overexpression of *ScPIP1* led to reduced MDA content and IL compared with that in the WT, suggesting that *ScPIP1* overexpressing transgenic plants may experience less membrane damage and lipid peroxidation under drought or salt conditions. Previous studies have reported that compared to the control, the overexpression of *TaAQP7* in transgenic tobacco (*Nicotiana tabacum* L.) exhibited lower levels of MDA and IL under drought stress [27] and the overexpression of *TaAQP8* in transgenic tobacco showed reduced MDA and IL under salt stress [28]. *MaPIP1;1* overexpressing transgenic *Arabidopsis* plants exhibited reduced MDA and IL under drought and salt stresses [35]. *OsPIP2;7* overexpressing transgenic rice (*Oryza sativa* L.) plants showed decreased IL under chilling stress [36]. In conclusion, these studies show that *PIP* acts as an important gene subgroup in reducing the MDA content and IL, accordingly decreasing membrane damage under various abiotic stresses. PIPs can facilitate water transportation through the membrane rapidly under optimum or drought and salt conditions, thereby maintaining a relatively healthy physiological status, which may contribute to reducing protein and lipid peroxidation, and decreasing membrane damage.

### 3.3. Response of ScPIP1 to Abiotic Stress Involved in Improving Osmotic Adjustment

The ability to maintain water balance is important for plants to resist drought and salt stresses, and PIPs play vital roles in the plant water balance process under water deficit conditions. We found that compared to the WT plants, *ScPIP1* overexpressing transgenic plants showed better growth and a higher survival rate under drought and salt conditions, suggesting a positive response of *ScPIP1* to water deficit. When plants experience drought and salt stresses, the accumulation of osmotic substances is considered a way to sustain osmotic adjustment. Proline, a compatible osmolyte, contributes to maintaining plant cell osmotic balance and enhancing cellular protection when plants suffered from stresses [37]. The overexpression of *ScPIP1* in transgenic plants increased the accumulation of proline compared to the WT plants under drought and salt stresses, indicating that *ScPIP1* may contribute to maintaining osmotic adjustment under water deficit conditions. We considered that the improved osmotic adjustment may also be related to the reduced membrane damage conferred by *ScPIP1* under drought and salt stresses.

## 4. Materials and Methods

### 4.1. Plant Materials, Growth Conditions and Abiotic Stress Treatments

Jojoba seedlings were obtained from the jojoba stem apex culture tube (SY03). Jojoba rooted seedlings were transferred to soil watered with Hoagland’s solution in growth chambers (28 °C; 110 μmol·m^−2^·s^−1^ light intensity; 16-h light/8-h dark cycle; 50% relative humidity). For the expression levels assay, six-week-old jojoba rooted seedlings in soil were watered with Hoagland’s solution supplemented with 30% PEG6000 for 0, 3, 6, 12, 24 or 48 h.

*Arabidopsis thaliana* ecotype Columbia (Col-0) was used as the WT control in the study and all transgenic lines were generated in the background of Col-0. Seeds were sterilized with 0.1% (*w*/*v*) HgCl_2_ for 10 min, washed 5 times with sterile water, sown on Murashige and Skoog (MS) medium (3% (*w*/*v*) sucrose, 0.7% (*w*/*v*) agar) and vernalized at 4 °C for 3 days in the dark. Ten-day-old seedlings were transferred to pots filled with a mixture of soil and sand (3:1) and grown in a chamber (22 °C; 110 μmol·m^−2^·s^−1^ light intensity; 16-h light/8-h dark cycle; 70% relative humidity). For germination test, the surface sterilized seeds were directly sown on MS medium supplemented with 0, 100, 200, 300 mM mannitol or supplemented with 0, 50, 100, 150 mM NaCl for 5 days. Radicle protrusion was used as a criterion for seed germination. For the root length assay, four-day-old seedlings were transferred to MS medium supplemented with 0, 100, 200, 300 mM mannitol or 0, 50, 100, 150 mM NaCl for 15 days. For the stress tolerance study, four-week-old plants in pots were unwatered or watered with 300 mM NaCl for 20 days. Then, photos were taken, and germination rates, root length and survival rates were calculated.

### 4.2. Cloning and Bioinformatics Analysis of Jojoba ScPIP1

An EST sequence (Accession Number: DV752738) of aquaporin was identified from drought induced suppression subtractive hybridization (SSH) cDNA library of eight-year-old female jojoba (SY03) [30]. By rapid-amplification of cDNA ends (RACE), the full-length cDNA of aquaporin was cloned from cDNA of jojoba leaves. For 5′ RACE, the forward primer sequence was 5′-tcagggggtcacattaaccc-3′ and the reverse primer sequence was 5′-acgagcacttcgcttagcatcag-3′. For 3′ RACE, the forward primer sequence was 5′-gaagatgattgcagcaccga-3′, and the reverse primer sequence was 5′-gcttccagccatctcagtatcag-3′. The EST sequences and the sequences of the 5′ RACE and 3′ RACE products were spliced together. Based on the sequences of the 5′ and 3′ ends, a pair of identical primers was designed (5′-atgggcaccatctccattgtca-3′ and 5′-cggaaacgaaacatcatacagtcgg-3′) to amplify the *ScPIP1* full-length sequence. The bioinformatics analysis of ScPIP1 was evaluated by DNAMAN software and BLAST (http://blast.ncbi.nlm.nih.gov/Blast.cgi).

### 4.3. qRT-PCR

Total RNA was extracted from jojoba leaves under various time treatments with 30% PEG6000 using the RNeasy Plant Mini Kit (Qiagen, Amsterdam, The Netherlands). Total RNA (1 μg) from each sample was converted into cDNA by reverse transcription using the RNA PCR Kit (TaKaRa) according to the manufacturer’s instructions. qRT-PCR was conducted on an ABI 7500 system (Applied Biosystems, New York, NY, USA) using TransStart™ Green qRT-PCR SuperMix Kit (TransGen, Beijing, China). *Sc16S rRNA* (GenBank Accession: AJ505848) was used as a reference gene to normalize the relative transcriptional abundance and to minimize different copy numbers of cDNA templates. The control sample was conferred a value of 1. All the data were calculated and analyzed from three independent samples based on the 2^-ΔΔ*C*t^ method [38]. The primers for *Sc16S rRNA* (5′-acaaggtagccgtactggaa-3′ and 5′-gccgagaaacgaaagaagac-3′) and *ScPIP1* (5′-cttccagccatctcagtatcag-3′ and 5′-agcacttcgcttagcatcag-3′) had high specificity according to the results.

### 4.4. Plant Transformation and Generation of Transgenic Plants

The *ScPIP1* ORF with PstI and AvrII restriction sites was amplified by specific primers (5′-aactgcagatggagggcaaggaggaggatg-3′ and 5′-cgcctaggcttagacttgaaggggatagcc-3′). The PCR products were cloned into the modified pCAMBIA1302-GFP expression vector to generate the 35S::*ScPIP1*-GFP fusion vector. The fusion vector was transferred into the *Agrobacterium tumefaciens* Smith & Townsend strain GV3101 and cultivated at a suitable concentration. Using the floral dip method, transgenic plants were generated [39]. Then, the seeds were selected by 40 mg/L hygromycin B on MS medium and the seedlings were identified by PCR amplification with specific primers (5′-tcacttcttggtccttctaccg-3′ and 5′-ttcaccttgatgccgttct-3′). Three T3 homozygous lines were generated and examined by PCR and semi-quantitative RT-PCR. *AtActin2* (GenBank Accession: NM_112764) was used as an internal control.

### 4.5. Rate of Water Loss

Forty integrated leaves were extracted from the four-week-old WT plants and transgenic lines, and then weighed immediately (fresh weight, FW). All samples were placed on respective open Petri dishes in a growth chamber (22 °C, humidity 45%) and were weighed every half hour (desiccated weights, DW) for 6 h. The water loss rates were calculated according to the formula: water loss rate (%) = (FW–DW) / FW × 100 [40].

### 4.6. MDA, IL and Proline Content Measurements

Four-week-old plants were not watered for 20 days or watered with 350 mM NaCl treatment for 15 d, then the leaves were collected to measure the MDA, IL and proline contents. The MDA content was measured using a malondialdehyde assay kit (Nanjing Jiancheng Bioengineering Institute, China). IL was determined based on the method described by Jiang and Zhang [41]. The samples were cut into strips and cultivated in 10 mL distilled water at room temperature for 8 h, and the initial conductivity (C1) was measured by a conductivity meter (DDBJ-350, Shanghai, China). Then the samples were boiled for 10 min. The conductivity (C2) was measured when the samples were cooled to room temperature. IL was calculated based on the equation: IL (%) = C1/C2 × 100. The proline content was measured using a proline assay kit (Nanjing Jiancheng Bioengineering Institute, China).

### 4.7. Statistical Analysis

The data are presented as means ± SD and were compared using the software Statistical Product and Service Solutions (SPSS) with one-way ANOVA followed by Duncan’s multiple range test at the significant level of *p* < 0.05.

## 5. Conclusions

**Figure 8:**
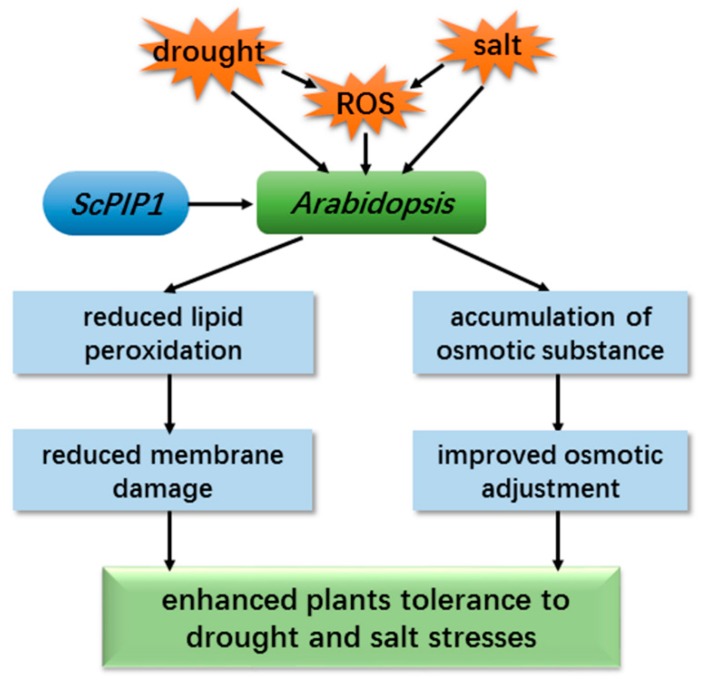
Effects of *ScPIP1* heterologous expression on tolerance to drought and salt stresses of *Arabidopsis*. Physiological responses to drought and salt stresses enhance the plant tolerance.

The findings of this study demonstrate the gene function of *ScPIP1* in response to drought and salt stresses. The overexpression of *ScPIP1* led to improved tolerance to drought and salt stresses by reducing membrane damage and improving osmotic adjustment (Figure 8). These results further illustrate the physiological mechanisms of transgenic plant resisting abiotic stresses and demonstrate the role of *PIPs* in decreasing membrane damage and maintaining osmotic balance. Furthermore, we must declare that the results from the experiments on transgenic *Arabidopsis* may not be the same when overexpressing *ScPIP1* in other species. Therefore, more studies on the function and role of *ScPIP1* in jojoba or other species are required.
